# Transient Limb Ischemia Alters Serum Protein Expression in Healthy Volunteers: Complement C3 and Vitronectin May Be Involved in Organ Protection Induced by Remote Ischemic Preconditioning

**DOI:** 10.1155/2013/859056

**Published:** 2013-12-02

**Authors:** Ting Pang, Yang Zhao, Nan-Rong Zhang, San-Qing Jin, San-Qiang Pan

**Affiliations:** ^1^Department of Anesthesiology, The Sixth Affiliated Hospital, Sun Yat-sen University, No. 26 Yuancunerheng Road, Guangzhou 510655, China; ^2^Department of Anatomy, Medical College of Jinan University, No. 601 West Huangpu Avenue, Guangzhou 510632, China

## Abstract

The protective mechanism underlying remote ischemic preconditioning (RIPC) is unclear. This study aims to verify whether the protein expression profile in the serum could be altered by RIPC and to detect potential protein mediators. Transient limb ischemia consisting of three cycles of 5-min ischemia followed by 5-min reperfusion was performed on sixty healthy volunteers. Serum samples were collected at 30 min before transient limb ischemia and at 1 hour (h), 3 h, 8 h, 24 h, and 48 h after completion of three cycles. Changes in the serum protein profile were analyzed by two-dimensional gel electrophoresis and proteins were identified by MALDI-TOF/TOF mass spectrometry. Fourteen differentially expressed proteins were identified and, respectively, involved in immune system, lipid binding and metabolism, apoptosis, and blood coagulation. Complement C3, vitronectin, and apolipoprotein A-I were further confirmed by western blotting, and the results showed that their contents decreased significantly after transient limb ischemia. It is concluded that transient limb ischemia alters the serum protein expression profile in human being, and that reduction of serum contents of complement C3 and vitronectin may represent an important part of the mechanism whereby RIPC confers its protection.

## 1. Introduction

Ischemic preconditioning (IPC), induced by exposing tissues to transient nonfetal ischemia prior to a prolonged ischemic insult [1], has been proved as a powerful strategy to attenuate ischemia reperfusion (IR) injury. This concept has been developed into remote ischemia preconditioning (RIPC), whereby transient tissue ischemia in one region or organ leads to subsequent protection in distant tissues or organs subjected to potentially lethal ischemia. Przyklenk and colleagues first showed that brief episodes of ischemia of the circumflex artery protect remote myocardium from subsequent sustained left anterior descending artery occlusion in the dog heart [2]. Furthermore, Kharbanda and colleagues conducted a clinical trial in humans and showed that contralateral forearm ischemic preconditioning induced by three cycles of arm ischemia and reperfusion is associated with diminished IR-induced endothelial injury [3]. From then on, this particular protocol has been shown to attenuate myocardial injury in patients with coronary heart disease [4–7]. Additionally, RIPC has been shown to have an early and late phase of protection [8].

However, the mechanism through which the protective signal is conveyed from the preconditioned limb to the remote organs is unclear, although the neural pathway [8], the humoral pathway [9], and systemic protective response have been proposed. The humoral pathway was suggested by the following studies. Dickson and colleagues showed that coronary effluent obtained from donor hearts subjected to brief preconditioning ischemia could reduce the infarct size in isolated buffer-perfused rabbit hearts [10]. A recent study by Shimizu and co-workers demonstrated that transient limb ischemia released unknown circulating factors which induced a potent protection against myocardial IR injury in Langendorff-perfused hearts and isolated cardiomyocytes in the same species, and this cardioprotection was transferable across species [11]. Our recent study found that the transfusion of plasma collected at late phase of RIPC into homogenic rats could improve the systolic blood pressure recovery during the reperfusion, suggesting that cardioprotective effect of transient limb ischemia was transferable via the plasma [12]. Experimental studies have attempted to identify humoral factors. Using proteomic methods, Lang and colleagues failed to identify a humoral mediator with a molecular weight of more than 8 kDa in rats subjected to remote ischemic preconditioning [13]. Serejo and colleagues speculated that thermolabile hydrophobic substances with molecular weights more than 3.5 kDa were cardioprotective factors in the effluent from preconditioned rat hearts [14]. However, most of the prior studies were performed on animals where circulating substances might vary with species and humoral mediators remained unknown.

We designed and conducted this study to investigate whether serum proteins could be altered by transient limb ischemia in human beings and to explore whether there existed any potential protein mediators in the serum that facilitate the protection induced by RIPC. In this study, transient limb ischemia was conducted in healthy volunteers and the approach of comparative proteomics was applied to identify serum proteins before and after transient limb ischemia. Proteins whose expressions were altered after transient limb ischemia were further studied and some of these proteins were validated by western blotting.

## 2. Materials and Methods

### 2.1. Subjects

We recruited sixty healthy volunteers and obtained written informed consent from them. Volunteers' characteristics were shown in Table 1. The study protocol was approved by the Ethics Committee of the Sixth Affiliated Hospital of Sun Yat-Sen University.

### 2.2. Induction of Transient Limb Ischemia

Transient limb ischemia was achieved by three cycles of ischemia and reperfusion, and each cycle consisted of 5-min ischemia followed by 5-min reperfusion of the nondominant arm. Ischemia and reperfusion were induced by a 12 cm-wide blood pressure cuff placed on the nondominant upper arm inflated to 200 mmHg for 5 min and then deflated for 5 min.

### 2.3. Sample Collection

At 30 min before transient limb ischemia and at 1 h, 3 h, 8 h, 24 h, and 48 h, respectively, after the the completion of three cycles of transient limb ischemia, blood (10 mL) was collected from the contralateral arm into tubes and was processed according to a standard operating procedure. The tubes were labeled and transported to the laboratory on ice within 15 min. The blood was centrifuged at 2500 rpm at 4°C for 10 min. Serum samples of each volunteer at all the time points were then collected, aliquoted, and stored at −80°C. Each serum sample underwent only two freeze/thaw cycles during all the following experimental protocols.

### 2.4. Two-Dimensional Gel Electrophoresis (2-DE)

Samples of 3 volunteers at all the time points were randomly selected from the samples of 60 volunteers to undergo the 2D-gel study. Each serum sample (1 mL) was processed by using reagents provided by the commercial ProteoMiner Protein Enrichment Kits (Bio-Rad) to decrease high-abundance proteins and to enrich low-abundance proteins. After enrichment, serum was purified by ReadyPrep 2D Cleanup Kit (Bio-Rad). Protein concentration was determined by the Bradford assay with the BSA standard (Bio-Rad).

A purified serum sample containing 150 *μ*g protein was diluted into 182 *μ*L with rehydration buffer (7 mol/L Urea, 2 mol/L Thiourea, 2% CHAPS, 1% DTT, and 0.2% Bio-lyte with pH 3–10) and then loaded to an 11-cm immobilized pH gradient (IPG) strip (Bio-Rad) followed by passive rehydration for 16 h. Isoelectric focusing (IEF) was performed at 250 V for 3 h followed by linear increase of 250–7000 V for 3 h, then at 8000 V for 7 h, and finally at 500 V for 30 min in the Bio-Rad PROTEAN IEF cell. IPG strips were equilibrated with reducing equilibration buffer (6 mol/L Urea, 50 mmol/L Tris-HCl pH 8.8, 20% (v/v) glycerol, 2% (w/v) SDS, and 2% (w/v) DTT) for 15 min followed by equilibration with an alkylating equilibration buffer (6 mol/L Urea, 50 mmol/L Tris-HCl pH 8.8, 20% (v/v) glycerol, 1% (w/v) SDS, and 2.5% (w/v) IAA) for 15 min. The strips were then placed in the well of 12% SDS-PAGE gels and sealed with 0.5% (w/v) agarose. Separation of the proteins in the second dimension was performed by 12% SDS polyacrylamide gel electrophoresis. The process began at 120 V for 30 min and continued at 150 V until tracking dye reached the bottom of the gel in a Protean Plus Dodeca cell (Bio-Rad). The gels were then fixed with the fixing solution (10% methanol plus 7% acetic acid in water) and stained with SYPRO-Ruby (Bio-Rad) for 16 h in the dark.

### 2.5. Protein Identification

Stained gels were scanned by densitometric scanning (Typhoon-9200, Amersham Company, Sweden) and the scanned images were exported into Image Master 2D Elite 5.0 software (Amersham Biosciences, Buckinghamshire, UK) for analysis. Differentially expressed protein spots in the gels, which were the spots expressed more than 1.5-fold differences in expression level as compared with the samples before transient limb ischemia at any time point after transient limb ischemia, were excised. The excised spots were immediately washed in redistilled water and then washed in 50% (v/v) acetonitrile in 100 mmol/L amine carbonate and then digested with 20 *μ*g/mL trypsin (Roche, Swiss). The extracted peptides were purified by ZipTip pipette tips with *μ*-C18 Resin (Millipore) and analyzed with tandem time-of-flight 4800 MALDI-TOF/TOF mass spectrometry (Applied Biosystems/MDS Sciex, Toronto, ON, Canada). Protein identification was performed by searching the MS/MS spectra against the international protein index (IPI) database, using a local MASCOT search engine (v2.1, Matrix, London, UK) on a global proteome server (v3.6, Applied Biosystems, Foster City, CA, USA). The database searches were performed using the following parameters: a maximum of 1 missed cleavage, variable modifications of methionine oxidation and cysteine carboxyamidomethylation, and precursor-ion mass tolerance of 0.2 allowed. *Homo sapiens* were selected as the search species. A protein identification was defined when its peptide had confidence interval value more than 95%.

### 2.6. Western Blotting

Based on their functional relevance and potential significance with regard to IR injury and organ protection, three proteins of complement C3, vitronectin, and apolipoprotein A-I (apoA-I) were selected to undergo western blotting. Original serum samples from all sixty volunteers were analyzed by western blotting to confirm expressions of the three proteins. Protein concentration was determined by the Bradford assay with the BSA standard (Bio-Rad), and then equal amounts of total protein (25 *μ*g) were loaded on 10% PAGE gels with 5% stacking gels. The gels underwent electrophoresis at 60 V for 30 min and 100 V for the duration of the run in running buffer (25 mmol/L Tris, 192 mmol/L glycine). The proteins in the gels were then transferred to polyvinylidene difluoride (PVDF) membranes (Millipore) at 100 V for 100 min in ice-cold transferring buffer (25 mmol/L Tris, 192 mmol/L glycine, 20% (v/v) methanol). After that, PVDF membranes were treated by the MemCode Reversible Protein Stain Kit for PVDF membranes (Pierce, NY) to ensure successful transfer of proteins and to allow for accurate quantitation of protein load. The membranes were blocked in Tris Buffered Saline with Tween (TBS-T; 170 mmol/L NaCl, 50 mmol/L Tris, pH 7.4, 0.1% Tween) containing 5% nonfat milk for 1 h at room temperature. The membranes were then washed in TBS-T three times (15 min for each time) and incubated with antibodies for 10 h to 12 h at 4°C. The dilution ratio of the antibodies for target proteins was: complement C3 1 : 500 (Santa, sc-52629), vitronectin 1 : 500 (R&D, Clone 342603), and apoA-I 1 : 500 (Abcam, ab52945). Membranes were then washed three times (15 min for each time) in TBS-T. The secondary antibody for complement C3 and vitronectin was donkey anti-mouse IgG-HRP 1 : 2000 (Santa, sc-2318) and for apoA-I was donkey anti-rabbit IgG-HRP 1 : 2000 (Santa, sc-2317). Membranes were incubated with secondary antibody for 1 h at 26°C and then washed three times in TBS-T (15 min for each time). Protein blots in the membranes were visualized by enhanced chemiluminescence (ECL) and then exposed to X-ray films (Kodak, USA). Films were scanned into digital images. The primary densities of the band of complement C3, vitronectin at 75 kDa, vitronectin at 65 kDa, and apoA-I were measured by Quantity One 4.62 (Bio-Rad), respectively. Protein expression levels were presented as relative density. The primary band densities at each time point after transient limb ischemia were divided by the band densities before transient limb ischemia (base line) in the same gel. Then relative densities before transient limb ischemia (base) were 1 according to the normalization in all conditions.

### 2.7. Statistical Analysis

Quantitative data were analyzed with statistical package SPSS 16.0 (Chicago, IL). Data were presented as mean ± standard deviation of the mean. Repeated-measures analysis of variance (RMANOVA) was used for serial measurements. Statistical significances were evaluated at a two-tailed significance level of 0.05. This trial was registered with ClinicalTrials.gov, no. NCT01118000.

## 3. Results

### 3.1. Serum Proteomic Profile

**Figure 1:**
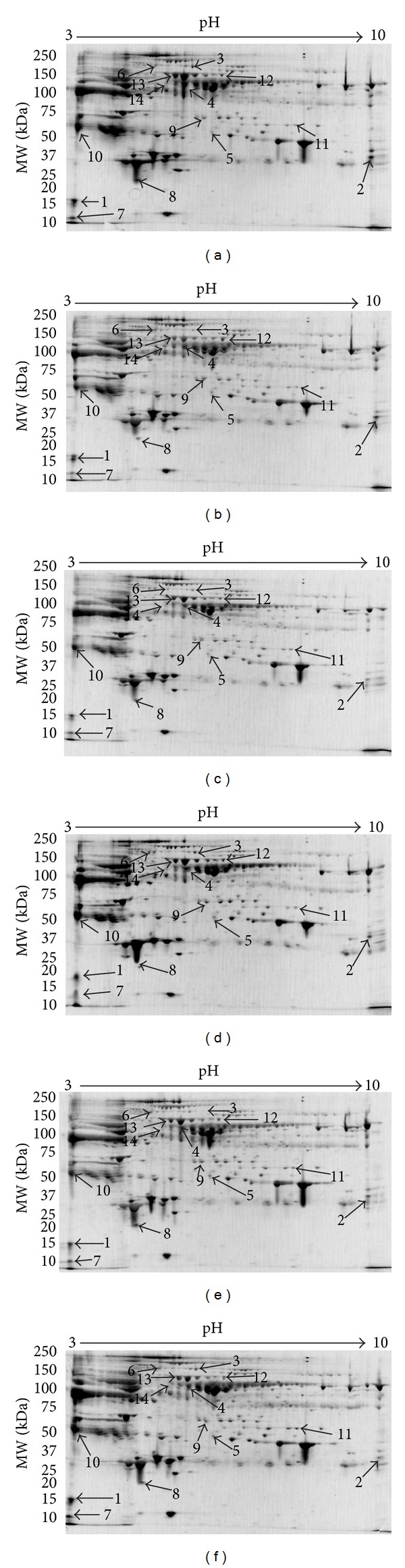
Representative images of SYPRO-Ruby-stained 2-DE gels. Representative images of SYPRO-Ruby-stained 2-DE gels at various time points ((a) before transient limb ischemia, (b) 1 h after transient limb ischemia, (c) 3 h after transient limb ischemia, (d) 8 h after transient limb ischemia, (e) 24 h after transient limb ischemia, and (f) 48 h after transient limb ischemia). The high-abundant proteins such as albumin and immunoglobulins were depleted from serum using the multiple-affinity column, as described in Section 2. Zoomed areas highlight typical spots (arrows) of the fourteen differentially expressed proteins. Changes in these spots' intensity among different time points are clearly visible. The spot numbers refer to proteins summarized in Table 2.

**Figure 2:**
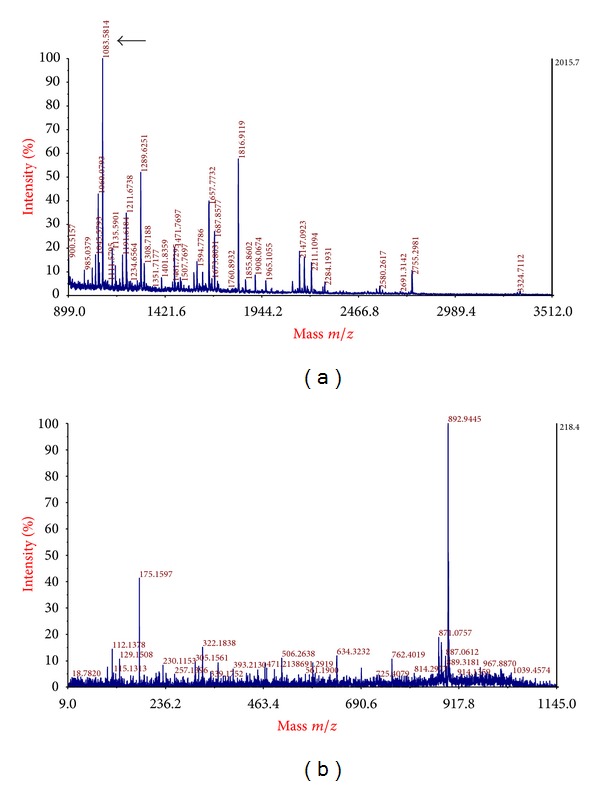
Peptide mass fingerprinting spectrum and a typical MS/MS map of complement C3. (a) Peptide mass fingerprinting spectrum of complement C3. The arrow indicates the peptide detected at *m/z* 1083.5814. (b) A typical MS/MS map of complement C3. The sequence of precursor at *m/z* 1083.5814 (arrow in (a)) was analyzed in this map.

Differentially expressed protein spots in the gels were identified as fourteen different proteins by MALDI-TOF/TOF mass spectrometry and database search (Figure 1). A typical MALDIMS spectrum and MS/MS map were shown in Figure 2. The fourteen identified proteins were classified based on gene ontology (GO) annotations (Table 2).

Among these fourteen proteins, seven proteins were related to immune response processes, including complement component 4B, complement C1q subcomponent subunit B, complement C3, C4b-binding protein alpha chain and ficolin-3 in complement pathway, vitronectin in immune response, and interalpha-trypsin inhibitor heavy chain H4 in acute phase response. Among the altered proteins, ficolin-3 and interalpha-trypsin inhibitor heavy chain H4 were upregulated, while the other proteins were downregulated. Besides, three proteins involved in lipid metabolic process were affected, namely, apoA-I and apolipoprotein J downregulated and apolipoprotein L1 upregulated. These proteins were also related to immune response. Then, two proteins involved in cell apoptosis were all upregulated, including desmoplakin and gelsolin. Finally, two proteins related to blood coagulation were also affected, antithrombin-III downregulated, and heparin cofactor 2 upregulated.

### 3.2. Changes of Complement C3, Vitronectin, and ApoA-I Validated by Western Blotting Analysis

**Figure 3:**
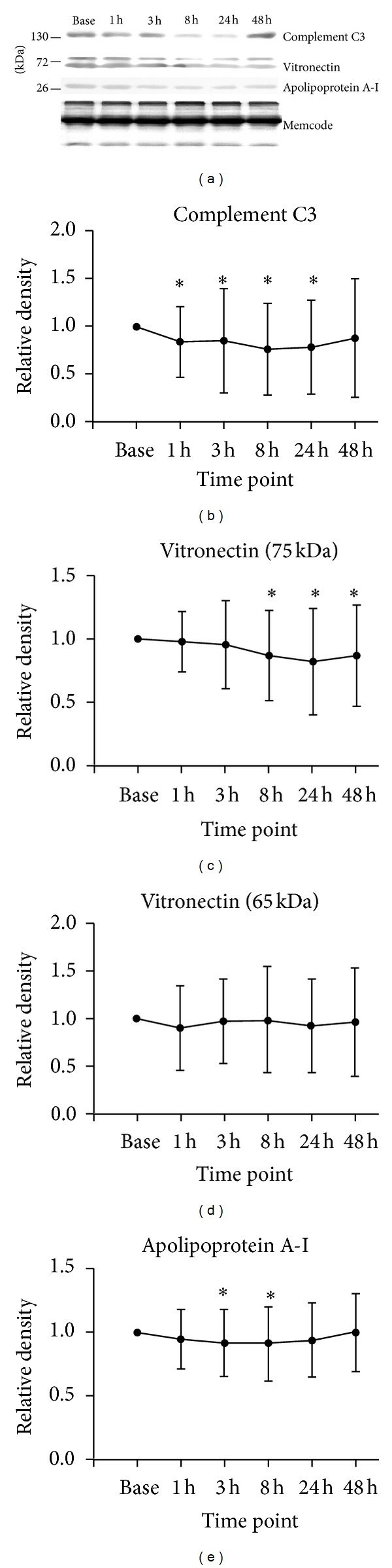
Western blotting data of complement C3, vitronectin, and apoA-I. The results were presented as mean ± SD. Error bars were SD. Repeated-measures analysis of variances was performed to evaluate statistical significance.*P < 0.05. (a) Representative western blotting images of serum samples from healthy volunteers obtained prior to transient limb ischemia (base) and at various time points (1 h, 3 h, 8 h, 24 h, and 48 h) thereafter. Memcode was shown to demonstrate equal protein loading. (b) Changes in the expression of complement C3 after transient limb ischemia compared with that before transient limb ischemia (*n* = 60). (c) Changes in the expression of vitronectin at 75 kDa after transient limb ischemia compared with that before transient limb ischemia (*n* = 60). (d) Changes in the expression of vitronectin at 65 kDa after transient limb ischemia compared with that before transient limb ischemia (*n* = 60). (e) Changes in the expression of apoA-I after transient limb ischemia compared with that before transient limb ischemia (*n* = 60).

The contents of the three proteins (complement C3, vitronectin, and apoA-I) in the volunteers' sera (*n* = 60) were decreased after transient limb ischemia. The decrease was significant at most of the time points as compared with the values before transient limb ischemia (Figure 3).

Baseline relative density of complement C3 was defined as 1. The reduction of complement C3 was significant at 1 h (0.84 ± 0.37 at 1 h versus 1; *P* = 0.002), 3 h (0.86 ± 0.54 versus 1; *P* = 0.049), 8 h (0.76 ± 0.48 versus 1; *P* = 0.000), and 24 h (0.78 ± 0.49 versus 1; *P* = 0.001) after transient limb ischemia but not at 48 h (0.88 ± 0.62 versus 1; *P* = 0.140, Figure 3(b)) after transient limb ischemia.

Baseline relative density of vitronectin (75 KDa) was defined as 1. The reduction of vitronectin (75 KDa) was significant at 8 h (0.87 ± 0.36 at 8 h versus 1; *P* = 0.013), 24 h (0.83 ± 0.43 versus 1; *P* = 0.004), and 48 h (0.87 ± 0.40 versus 1; *P* = 0.023) after transient limb ischemia but not at 1 h (0.98 ± 0.24 versus 1; *P* = 0.604) and 3 h (0.96 ± 0.35 versus 1; *P* = 0.409; Figure 3(c)) after transient limb ischemia.

Baseline relative density of vitronectin (65 KDa) was defined as 1. The reduction of vitronectin (65 KDa) was not significant at 1 h (0.90 ± 0.44 versus 1; *P* = 0.123), 3 h (0.97 ± 0.44 versus 1; *P* = 0.646), 8 h (0.98 ± 0.56 versus 1; *P* = 0.789), 24 h (0.92 ± 0.49 versus 1; *P* = 0.261), and 48 h (0.96 ± 0.56 versus 1; *P* = 0.646) after transient limb ischemia (Figure 3(d)).

Baseline relative density of apoA-I was defined as 1. The reduction of apoA-I was significant at 3 h (0.92 ± 0.26 at 3 h versus 1; *P* = 0.016) and 8 h (0.91 ± 0.29 versus 1; *P* = 0.026) after transient limb ischemia but not at 1 h (0.95 ± 0.23 versus 1; *P* = 0.119), 24 h (0.94 ± 0.29 versus 1; *P* = 0.11) and 48 h (1.00 ± 0.30 versus 1; *P* = 0.905) after transient limb ischemia (Figure 3(e)).

## 4. Discussion

There were hardly any studies conducted amongst healthy volunteers in order to detect clinically relevant protein mediators that facilitate the protective effects of RIPC. Isolated buffer-perfused animal hearts in vitro and animal models in vivo have been extensively used for investigating the protective effect and mechanism of RIPC. Although these models have been shown to be reproducible and efficient, isolated hearts could not reflect the effects of nervous and circulatory system in the whole body, and animal disease models cannot represent the complex human clinical setting very well. Therefore, conducting a controlled human study is a crucial translational step from bench to bedside. A recent human study by Hepponstall et al. found that the RIPC stimulus modified the plasma protein content in blood, but this study enrolled only five healthy volunteers [15].

In this study, we compared serum proteins profiles after transient limb ischemia with that before transient limb ischemia in healthy volunteers by a proteomic approach and showed that there existed changes of serum proteins induced by transient limb ischemia. The changed proteins were mainly involved in the inflammatory system and also involved in the lipid metabolic system, cell apoptosis, and coagulation system.

In the 2-DE results of our study, we observed that complement component 4B, complement C1q subcomponent subunit B, C4b-binding protein alpha chain, and complement C3 were all decreased after transient limb ischemia.

Furthermore, we confirmed the changes of three proteins of complement C3, vitronectin (75 KDa), and apoA-I after transient limb ischemia by western blotting. The downregulation of complement C3 could persist for one day or perhaps even longer, the content of vitronectin (75 KDa) decreased significantly at 8 h, 24 h, and 48 h after transient limb ischemia, and the content of apoA-I decreased significantly at 3 h and 8 h after transient limb ischemia.

The pathway through which RIPC protects organs is unclear, but three possible mechanisms have been suggested [16]. The neural hypothesis proposed that the remote preconditioned organs could release endogenous substances such as adenosine [17], bradykinin [18], or calcitonin gene-related peptide [19] which activated afferent neural pathway terminating at the heart to confer myocardial protection. On the other hand, the humoral hypothesis thought that these released endogenous substances or some other humoral factors were carried to the heart in the blood stream and recognized specific receptors in the myocardium to activate intracellular pathways of myocardial protection. Finally, RIPC could suppress inflammation and apoptosis, which was called systemic protective response [20]. Our results showed the changes of serum proteins after transient limb ischemia and these proteins mainly involved in inflammatory response, which may support the second and third hypothesis above mentioned.

The complement cascade has been shown to be a key mediator of IR injury [21, 22]. Inhibition of the complement system can improve the outcome of IR injury [23, 24]. In a rabbit model, Tanhehco and co-workers observed that ischemic and chemical preconditioning inhibited the upregulation of complement C1q, C1r, C3, C8, and C9 mRNA expression and the complement C3 and membrane attack complex protein expression caused by IR injury both in vivo and in isolated heart in rabbits [25, 26].

Complement C3 is the central molecule of the complement system, at which the classical, lectin, and alternative pathways converge. Complement C3 is also associated with myocardial infarction, and it is more significant than any other traditional risk factors [27]. We selected complement C3 for further validation by western blotting and showed that the complement C3 expression in human serum significantly decreased from 1 h to 24 h after transient limb ischemia. A study by Zheng and colleagues demonstrated that siRNA solution containing siRNAs targeting tumor necrosis factor *α*, complement C3, and Fas genes could decrease cardiac IR injury, protect cardiac function, and prolong graft survival in heart transplantation [28]. Mocco and co-workers used mice deficient in selected complement proteins (C1q, C3, and C5) to evaluate which complement subcomponents contribute to cerebral IR injury and demonstrated that only C3−/− mice experienced significant neuroprotection [29].

Vitronectin is a multifunctional glycoprotein present in plasma, extracellular matrix, and blood platelets. It is found in two molecular forms in human blood, which are a single chain (75 kDa) and a clipped form of two chains (65 and 10 kDa) [30]. In our study, we also observed the vitronectin fragment located at ~10 kDa by 2-DE and the vitronectin doublet located at 65~75 kDa by western blotting. Vitronectin has been implicated as a regulator of many diverse physiological processes [31]. Ekmekci and colleagues showed that plasma vitronectin levels in patients with coronary artery diseases were significantly increased and positively correlated with the extent of diseases [32]. Yamani and colleagues observed that myocardial ischemic injury after cardiac transplantation was associated with upregulation of vitronectin receptor (integrin*α*V*β*3) [33]. Using data from a randomized, placebo-controlled trial of abciximab in patients undergoing percutaneous coronary intervention, Derer and co-workers found that serum concentration of vitronectin was an independent risk factor for adverse cardiovascular events [34].

ApoA-I is the major protein of high-density lipoprotein. Besides its role in cholesterol metabolism, it possesses antiatherosclerotic, antioxidant, anti-inflammatory, and antithrombotic activities [35, 36]. A recent study by Shi and Wu showed that apoA-I reduced IR-induced inflammatory responses, decreased renal microscopic damage, and improved renal function [37]. Our results may implicate that apoA-I is not involved in the protective mechanism of RIPC.

This study was designed as a self-control study to confirm whether the composition of serum proteins was changed after transient limb ischemia in human beings. Time points for blood sample collection were selected in consideration of early and late phase of protection induced by RIPC. The early phase lasts for up to 3 h after ischemic preconditioning, whereas the late phase starts at 12–24 h after ischemic preconditioning [8]. Our study suggested that reduction of complement C3 and vitronectin expression may be involved in the mechanism by which preconditioning salvage tissues or organs subsequently subjected to IR, although they are likely to reflect changes induced by the preconditioning mediators. Further study is needed to find by which way these proteins are altered by transient limb ischemia and confirm whether these altered proteins actually protect organs.

It should be noted that it is very complicated to elucidate the mechanism of RIPC. Nevertheless, this study just finds some changes of serum proteins at limited time points which should serve to facilitate future extensive work aimed to elucidate the RIPC protective mechanism.

## Figures and Tables

**Table 1:** Healthy volunteers' characteristics*.

Age (years)	22 (1.8)
Male	30 (50%)
Mean arterial pressure (mmHg)	85 (9)
Pulse (bmp^†^)	75 (12)
Pulse oxygen saturation (%)	99 (1)
Body mass index (Kg/m^2^)	20.49 (2.30)

*Data are mean (SD) or counted number (%).

^†^bmp: beats per minute.

**Table 2:** Differentially expressed proteins in sera after RIPC compared with that in sera before RIPC.

Spot^a^	Protein name	Accession number^b^	Protein MW (Da)	Protein PI^c^	Sequence coverage^d^ (%)	Fold change^e^	Protein score^f^
(i)	*Immune system process *						
1	Complement component 4B	IPI00887154	192627.5	6.89	2	↓1.95^1 h^	158
2	Complement C1q subcomponent subunit B	IPI00477992	26704.5	8.83	22	↓2.73^3 h^	84
3	Complement C3	IPI00783987	187029.9	6.02	15	↓2.23^1 h^	121
4	C4b-binding protein alpha chain	IPI00021727	66989.4	7.15	9	↓1.51^1 h^	71
5	Ficolin-3	IPI00419744	31657.4	6.36	18	↑1.52^1 h^	107
6	Interalpha-trypsin inhibitor heavy chain H4	IPI00896419	103293	6.51	18	↑2.51^1 h^	97
7	Vitronectin	IPI00298971	54271.2	5.55	4	↓1.52^1 h^	70

(ii)	*Lipid metabolic process *						
8	Apolipoprotein A-I	IPI00021841	30758.9	5.56	25	↓3.85^1 h^	79
9	Apolipoprotein L1	IPI00514475	43946.9	5.6	28	↑1.68^1 h^	88
10	Apolipoprotein J	IPI00291262	52461	5.89	20	↓1.74^24 h^	219

(iii)	*Apoptosis *						
11	Desmoplakin	IPI00013933	331568.7	6.44	8	↑1.62^48 h^	67
12	Gelsolin	IPI00026314	85644.2	5.9	16	↑2.21^1 h^	131

(iv)	*Blood coagulation *						
13	Antithrombin-III	IPI00032179	52657.8	6.11	23	↓2.01^1 h^	180
14	Heparin cofactor 2	IPI00879573	57034.2	6.41	17	↑2.32^1 h^	64

^a^Spot number refers to Figure 1.

^b^Accession number from IPI (International Protein Index) database of matched proteins.

^c^PI refers to isoelectric point.

^d^Percent of number of observed amino acids in sequence length (%).

^e^“↓” means downregulation, and “↑” means upregulation. Superscripts such as “1^ ^h” represented the time point when the fold change reached maximum. The fold change in the table was the maximum change among the different time points.

^f^Combined scores of all observed mass spectra matched to amino acid sequences used for protein identification.
